# Impact of interseismic deformation on phase transformations and rock properties in subduction zones

**DOI:** 10.1038/s41598-019-56130-6

**Published:** 2019-12-20

**Authors:** Sebastian Cionoiu, Evangelos Moulas, Lucie Tajčmanová

**Affiliations:** 1 Heidelberg University, Institute of Earth Science, Heidelberg, Germany; 2 Johannes Gutenberg University, Institute of Geosciences, Mainz, Germany

**Keywords:** Petrology, Tectonics, Geodynamics, Structural geology

## Abstract

Phase transformations greatly affect physical properties of rocks and impose a first-order control on geodynamic processes. Under high deformation rates, rheological heterogeneities cause large spatial variations of stress in materials. Until now, the impact of higher deformation rates, rock heterogeneity and stress build up on phase transformations and material properties is not well understood. Here we show, that phase transitions are controlled by the stress build-up during fast deformation. In a deformation experiment (600 °C, 1.47 GPa), rock heterogeneity was simulated by a strong elliptical alumina inclusion in a weak calcite matrix. Under deformation rates comparable to slow earthquakes, calcite transformed locally to aragonite matching the distribution of maximum principal stresses and pressure (mean stress) from mechanical models. This first systematic investigation documents that phase transformations occur in a dynamic system during deformation. The ability of rocks to react during fast deformation rates may have serious consequences on rock rheology and thus provide unique information on the processes leading to giant ruptures in subduction zones.

## Introduction

In reacting rocks, phase transformations have a great influence on rheology through variations in mineral assemblages, mechanical properties, the presence of fluid or a change in grain size^1^^,^^2^. Rock microstructures are a result of coupled chemical and mechanical processes^3^. Previous studies have demonstrated that material heterogeneity can lead to the development of heterogeneous stress and pressure (mean stress) distributions^4,5^. On the long term, large scale geological processes are commonly assumed to be slow and stress variations on geological timescales negligible. However, as recently documented by geophysical methods, transient periods with higher deformation rates (10^−9^–10^−10^s^−1^) and stress build-up are more frequent than previously thought^6,7^. These fast deformation events can result in large earthquakes with direct societal consequences. For example, slow earthquakes may play an important role in the more damaging earthquake cycles in subduction zones. These processes can take place along the megathrust and other planes of weakness in response to loading, releasing low frequency seismic waves^6^. Slow-slip events during subduction were inferred from accurately measured crustal movements and it was shown that these occur over periods of hours to weeks^8,9^. Understanding processes during fast deformation events depends on identifying material behavior and its rheology. While the effects of high deformation rates (10^−5^–10^−6^s^−1^) on phase transformations were previously studied in nominally homogeneous experiments^10–12^, the processes in heterogeneous materials remain unclear. Interestingly, the time scale for these slow earthquakes (~10^−9^s^−1^) approaches experimental strain rates (~10^−6^s^−1^) which opens a new horizon for designing deformation experiments on reactions in rheologically heterogeneous materials.

**Figure 1:**
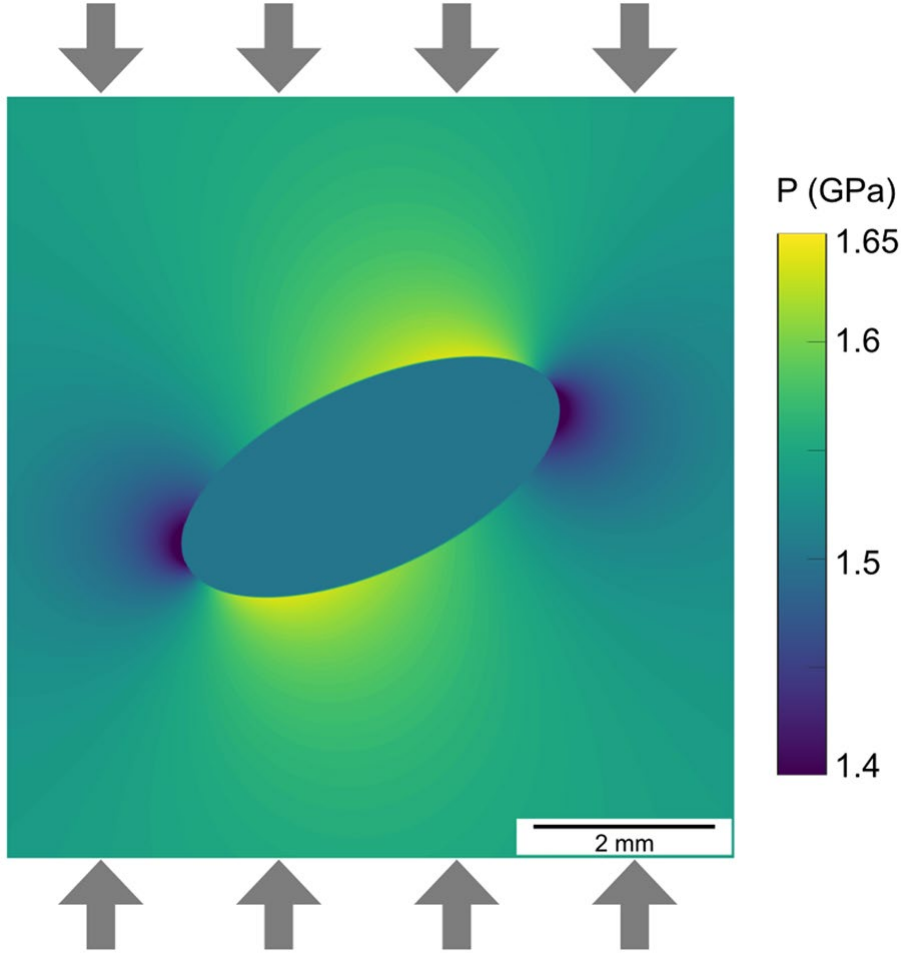
Analytical solution for a strong elliptical inclusion in a weaker matrix under vertical compression (viscosity ratio 100:1). This calculation follows an analytical solution^4^ and shows the distribution of the pressure (mean stress) field resulting from a material heterogeneity under stress. The far-field differential stress is 0.14 GPa at 1.47 GPa confining pressure. The ellipse axis ratio is 2.2: 1.

Rocks are commonly heterogeneous, composed of minerals with different physical properties such as viscosity. The variations of viscosity develop stress variations and can trigger mechanical instabilities during deformation^13–16^. The effect of elliptical heterogeneities on the stress field of a deforming viscous material has been investigated by analytical solutions^4,17^. These models show that material heterogeneity, expressed as viscosity heterogeneity, can lead to the development of stress and pressure spatial variations around a strong inclusion (Fig. 1). However, such models do not involve reacting materials and thus they do not consider the interplay between phase transformations and deformation.

## Experimental Setup

**Figure 2:**
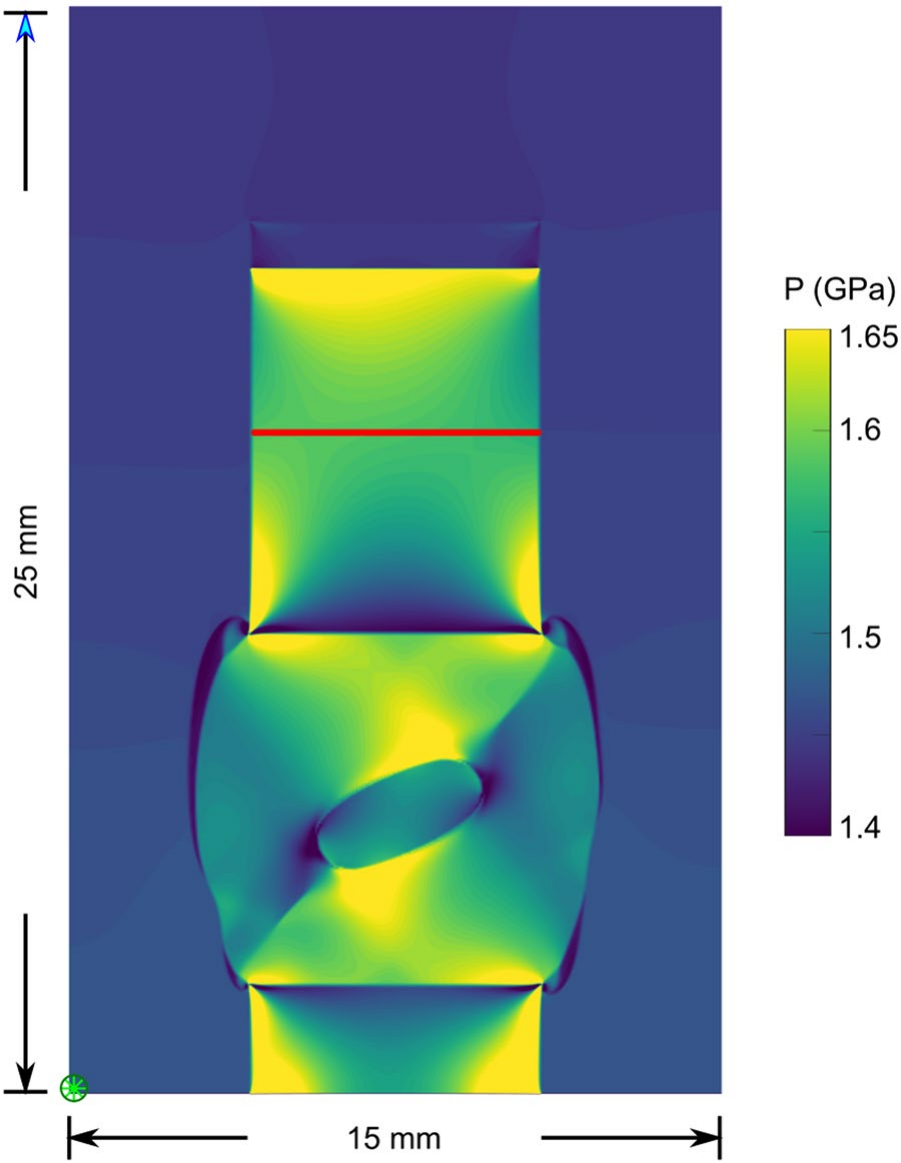
Numerical model for the experimental configuration. The image shows the pressure state of the model after 2.9 mm vertical shortening (initial length 11 mm). The geometry replicates the experimental configuration. The red line indicates the region from which the deformation stress (σ_yy_) was determined. The representative value of the confining pressure (P_conf_; mean stress in the confining medium) was obtained from the lower-left corner of the model at the point indicated by the green asterisk (*).

Here, we designed and performed a coaxial deformation experiment on rheologically heterogeneous, viscous materials to investigate the effect of local stress variations, resulting from fast deformation rates (here 10^−6^s^−1^), on mineral transformations. We took an inspiration from and validated the results against the analytical solutions of a strong ellipse within a weak matrix^4,17^. In addition, we performed numerical models that corroborate the first-order predictions of the stress field from analytical solutions (Fig. 2). The results of these mechanical calculations were, for the first time, compared to the experimentally produced phase distribution.

We conducted a pure shear viscous deformation experiment close to the calcite-aragonite (CaCO_3_) phase transition in a Griggs-Type deformation apparatus. The error of confining pressure and differential stress measurements is ca. 0.03–0.05 GPa (see Methods for details and references). The sample material was dried cold-pressed natural calcite-powder (63–125 μm grain size) with added 0.1% of H_2_O. We introduced a viscosity heterogeneity by embedding a strong (i.e. more viscous) and non-reactive elliptical alumina (Al_2_O_3_) inclusion within the calcite powder. The inclusion was initially tilted by 45° relative to the axial-shortening direction. The experiment was performed at 600 °C to avoid kinetic limitations, and a confining pressure of 1.47 GPa, i.e. 0.08–0.1 GPa below the phase transition of calcite to aragonite (see Supplementary Fig. S1). This confining pressure was chosen in a way that the expected maximum principle stress reached the stability field of aragonite, while the confining pressure remained in the calcite stability field. The total axial displacement was 3 mm at a constant rate of 10^−8^ m/s (i.e. the duration of deformation was 80 h) and produced a barrelled cylinder of 7.4 mm in length.

**Figure 3:**
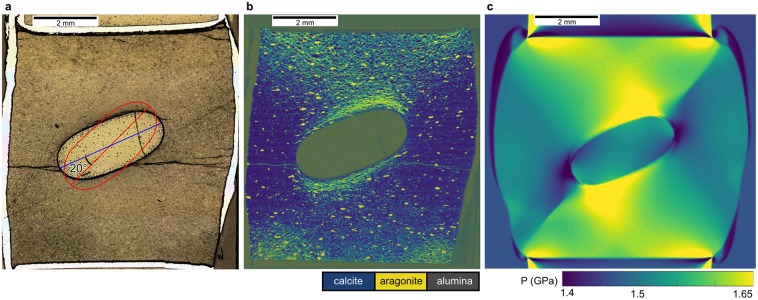
Comparison of experimental and numerical modelling results. (**a**) Reflected light photomicrograph of the recovered sample 010SC. The blue trace shows the final orientation of the ellipse’s long axis. The red trace indicates the initial position at 45° relative to the deformation direction. The platinum capsule corresponds to the white material around the sample. (**b**) Raman spectroscopy map of sample 010SC. The starting material, calcite, is shown in blue, while yellow indicates the formation of aragonite (see text for details). (**c**) Pressure distribution in the numerical model after 30% axial strain (close up view of Fig. 2). The modelled pressure pattern matches the distribution of the experimentally produced high pressure polymorph aragonite.

During deformation, a sample-scale (bulk) finite strain rate of 10^−6^ s^−1^ and a peak differential stress of 0.14–0.18 GPa was reached (run-data and corrections are shown in Methods and Supplementary Figs. S2–S5). After the experiment, the sample was cut in the plane defined by the σ_1_ direction and ellipsoid long axis, which is also the modelled orientation. We used reflected light microscopy (Fig. 3a) to determine deformation patterns and Raman spectroscopy mapping (step-size 12 μm) to resolve the phase distribution (Fig. 3b, Supplementary Figs. S6 and S7). The data of an undeformed experiment (009SC) are shown in the Supplementary Material for comparison (Figs. S8 and S9).

## Numerical Modelling Results

In order to estimate the local variations of stress and strain within the experiment, we used a 2D finite-difference numerical model^18^. This model is a geometrical 1:1 representation of a cross section through the experiment and resolves the stress distribution between deformation pistons, sample and ellipse (Fig. 2). For the deforming weak material, a viscous power-law rheology for calcite was used^19^. A linear viscosity was used for the strong inclusion (equal to the upper and lower pistons). Deformation was achieved by imposing a constant displacement rate in the upper alumina piston (see Methods and Supplementary Fig. S5).

We validated the numerical model and input parameters by comparing them to the experimentally measured bulk stress. Therefore, we analysed the experimental and modelling results at two spatial scales: (1) the bulk stress values that were extracted from the model (see location in Fig. 2) and compared to the experiment run-data and (2) the modelled deformation patterns that were compared to the sample deformation. To allow direct comparison, we used the same displacement rate and confining pressure as in the experiment. In addition, we used the exact shape of the experimental ellipsoid cross-section and its initial orientation as model input. Both, bulk stresses and deformation patterns were in good agreement between model and experimental data (Supplementary Figs. S2–S4). Finally, we compared the phase distribution in the deformed sample to the stress, pressure and strain distribution in the mechanical models.

## Experimental Results

The deformation in the experiment lead to slightly skewed, barrel-shaped sample, while the ellipsoid rotated by ca. 23° relative to its original orientation (Fig. 3a). Within 80 hours, the starting material, calcite, preferentially transformed to its high-pressure polymorph aragonite mainly at the ellipsoid faces oriented perpendicular to the compression direction and close to the piston-sample interface (Fig. 3b). While some aragonite formed in the triangular area in-between piston and ellipsoid, the phase transition is hardly occurring between the ellipsoid and the outer jacket sides. The hydrostatically-calibrated phase transitions from calcite to aragonite occurs at 1.55 GPa at 600 °C (Supplementary Fig. S1). As shown in Fig. 3b,c, the distribution of fine-grained aragonite, quantitatively fits with the modelled local pressure distribution. The distribution of bigger aragonite grains (>0.1 mm) is also in agreement with the pressure pattern as shown in the Supplementary Fig. S7.

## Discussion

The comparison of the modelled stress and strain patterns in the deforming material (calcite) and the experimentally produced phase transition distribution shows a direct correlation between the modelled local pressure (mean stress) and the phase transformation pattern all over the sample (Fig. 3b,c). Aragonite (the high-pressure polymorph) in the experiment is more abundant in regions where the numerical model predicts higher pressures locally. Around the ellipsoid the modelled pressure agrees with the pressure distribution given by the analytical solution^4^ (Fig. 1). This agreement shows that stress heterogeneities caused by the geometry of the sample assembly (e.g. stress concentrations at corners etc.) play a minor role in the vicinity of the ellipse at the centre of the sample. The numerical model for the experimental configuration shows that local pressure (mean stress) can exceed bulk σ_1_ or fall below bulk σ_3_ depending on the mechanical configuration. Thus, the locally resolved knowledge of the stress-state is essential to better understand the bulk deformation and material property changes.

The development of significant stress variations in the model and the experiment is caused by high deformation rates. The results document that such deformation rates can lead to an increase of mean stress in the sample and local stress perturbations (positive and negative) in the order of 0.1–0.3 GPa for the case of calcite. These variations in stress and pressure were responsible for the fast transformation of calcite to aragonite, a higher-pressure polymorph that has different physical and mechanical properties. Interestingly, when composite materials are considered, the effective mechanical behaviour is grossly influenced by the individual phase properties as well as their spatial distribution^2,20^.

Our results show that the phase transformation was not completed, as evidenced by the partial retainment of calcite grains in association with aragonite. However, the locally elevated pressure could be responsible for the higher degree of overstepping above the calcite/aragonite transition as documented by higher amounts of aragonite in those regions (see Fig. 3 and Supplementary Fig. S7). This indicates that the local mechanical configuration significantly affects the distribution of the reactants prior to the achievement of mineral equilibrium.

The effect of fast deformation rates on mineral transformation is not limited to experimental conditions only. In fact, recently discovered fast deformation processes that occur in subduction zones^6,8^ require deformation rates that are approaching our experimental conditions. Therefore, high stress that develops during subduction processes may be accompanied by mineral reactions and mineral transformations that affect the effective mechanical behaviour of heterogeneous rocks.

During the periods of inter-seismic loading of the lithosphere, differential stress can be in the order of 0.1 GPa^21^. Our results show that these values of differential stress can trigger mineral reactions and phase transformations locally. Such mineral transformations and the accompanying change in material properties during the deformation thus have a dramatic impact on the stability and style of deformation, as well as on the degree of coupling of the subduction interface.

## Methods

### Experiments

The deformation experiment was carried out in a Griggs-Type deformation apparatus at 600 °C and 1.47 GPa confining pressure. For our experimental set-up, we estimate the error in confining pressure to be +0.01/−0.05 GPa. For differential stresses the error is estimated to be ±0.03 GPa due to the weak, yet solid confining medium (Potassium-Iodide)^12,22,23^. The vertical temperature gradient along this sample is assumed to be ca. 20–30 °C and no horizontal temperature gradient is assumed across the sample^24,25^. In order to have a quantitative estimate of the temperature gradients, we calculated the steady-state temperature distribution in two dimensions following the experimental geometry (Supplementary Fig. S8). The constant displacement rate of 10^−8^ m/s corresponds to a (temporal and local) averaged strain rate of 10^−6^s^−1^.

The sample material was calcite-powder (sieved to 63–125 μm, not washed) which we obtained from crushing a high purity icelandspar single crystal (from Helgustadir, Iceland). The mineralogy of the single crystal was verified by Raman spectroscopy. The powder was placed in a 0.15 mm thick, welded Pt jacket. The alumina (Al_2_O_3_) ellipsoid had a 3.75 mm long axis and 1.65 mm short axis and was initially tilted by 45° relative to the displacement direction. To obtain this configuration, the powder was pre-compressed at a 45° angle; a small groove was introduced into this surface, so that the ellipsoid could be positioned and then further covered with calcite powder. For 1000 mg of calcite, 0.8 μl of H_2_O were added. The capsule was then cold-pressed at 0.05 GPa before closing. The assembly was hot pressed for 14 h at 1.47 GPa and 600 °C before deformation. After the experiment, the sample was quenched from 600 °C to 200 °C at a rate of 20 °C/s to assure a good preservation of reacted phases.

The ellipsoid long-axis orientation was marked on the jacket for later cutting. The length of the cold-pressed sample was 13.4 mm and the measured total axial displacement was 3 mm. The sample length after compaction during loading and hot-pressing, and deformation was 7.4 mm. The inferred length of the sample after compaction (i.e. before deformation) is 10.4 mm.

The pressure of the phase transition was previously calibrated on this machine in hydrostatic experiments – to ensure that σ_3_ was in the calcite field. Mechanical data (deformation stress and displacement) for the experiment was corrected for machine compliance, machine friction during run-in and increasing sample area. The raw data and resulting corrected stress-time and stress-strain curves (two correction procedures are discussed) are portrayed in the Supplementary Figs. S2 and S3.

### Numerical modelling

To model the stress and pressure distribution in the deformed sample, we solved the mechanical problem numerically. We solve the Stokes equations for slow viscous, incompressible flow with a nodal resolution of 301 × 501 grid points. The code uses the MATLAB® direct solver and employs a staggered grid for discretisation^18^. Following ref. ^18^, the marker in cell method is used for model advection. Temperature is assumed to be constant during deformation.

Boundary conditions were set to free slip at the sides and top of the model, while the bottom was set to no-slip. An internal zone of constant displacement rate was set in the upper alumina piston (constant displacement rate in vertical direction, no displacement in horizontal direction) in order to control the bulk deformation rate of the sample region. Time steps were set to a maximum of 450 s. After 700 calculation steps a strain of ε = 0.33 was reached.

The rheology of the deforming material (calcite) was modelled using a power-law viscosity^19^ (Carrara marble, regime 2), and recalculated to strain-rate dependent effective viscosity (Eq. (1)) using the relationships between stress and strain invariants and experimentally derived values described in ref. ^26^:

1 $${\eta }_{eff}=\frac{1}{{2}^{(n-1)/n}{3}^{(n+1)/2n}}\cdot \frac{1}{{A}^{1/n}{{\dot{\varepsilon }}_{II}}^{(n-1)/n}}\cdot \exp (\frac{Q}{nRT})$$

where, η_eff_ is the effective viscosity; ${\dot{\varepsilon }}_{{\rm{II}}}$ is the second strain rate invariant, R is the gas constant, n is the stress exponent (n = 7.6); T is temperature (T = 873 K); Q is the activation energy (Q = 418.4 kJ/mol) and A is a constant (A = 10^−4.5^ × 10^(5(−n))^ Pa^−n^ s^−1^); all in SI units.

The viscosity for the other materials was assumed to be constant: for alumina 10^16^ Pa s (pistons and ellipse are equal) and for salt 3 × 10^11^ Pa s.

### Raman spectroscopic mapping

Raman mapping was carried out using WITec Alpha 300R microscope and UHTS 300 Spectrometer VIS-NIR at Heidelberg University. The x-y stage operated at a step-size of 12 μm and a 50x objective was used. The grating was set to 1200 grooves/mm. The excitation laser wavelength was 531.98 nm. The laser intensity at the sample was 50 mW, which yields clearly distinguishable spectra with 0.25 s acquisition time (Supplementary Fig. S6). For the map interpretation at each pixel, the spectra were analysed and assigned to calcite (peak at 282 cm^−1^ ^27^), aragonite (peak at 205 cm^−1^ ^28^) or a mix with colours accordingly ranging from blue to yellow.

## Supplementary information

Supplementary information 

## Data Availability

Unprocessed experimental data and details on numerical modelling are available in the Supplementary Materials. Further requests for materials and codes should be addressed to L.T. (lucataj@gmail.com) or S.C. (sebastian.cionoiu@geow.uni-heidelberg.de).
