# Supplementary material for: Impact of interseismic deformation on phase transformations and rock properties in subduction zones
Source: Sci Rep. 2019 Dec 20;9:19561. doi: 10.1038/s41598-019-56130-6 (PMC6925112; doi:10.1038/s41598-019-56130-6)
Supplement: Supplementary file 1 — Supplementary information [file 41598_2019_56130_MOESM1_ESM.pdf]

SUPPLEMENTARY INFORMATION

Impact of interseismic deformation on phase transformations and rock properties in subduction zones

Sebastian Cionoiu, Evangelos Moulas, Lucie Tajčmanová

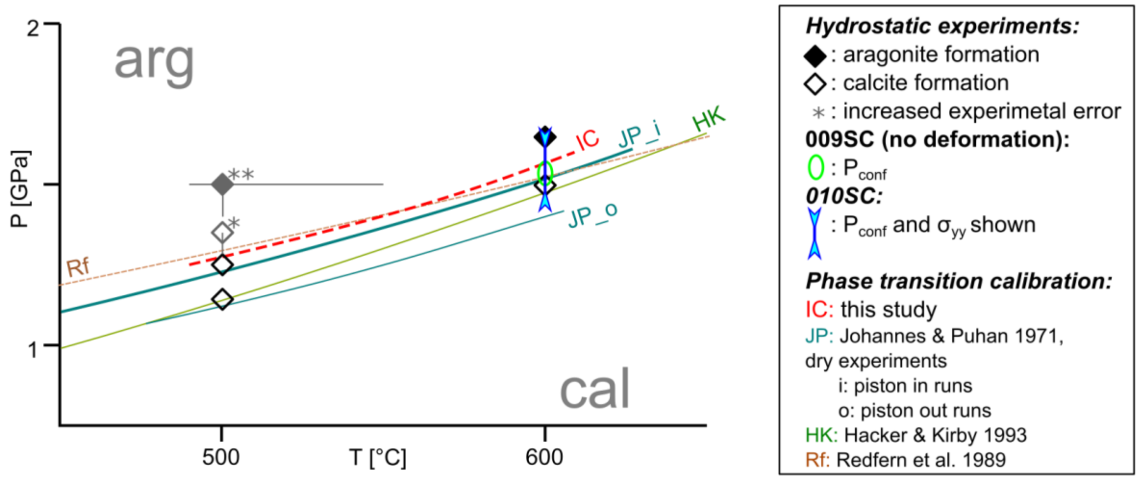

Fig. S1. *P*-*T* diagram showing the calcite-aragonite transition. Hydrostatic experiments were performed to define an internally consistent phase transition pressure (IC) for the Griggs apparatus used for this study. In the two experiments marked by asterisks (*), the error bars depict increased experimental error due to measurement uncertainties (thermocouple failure, increased friction). An undeformed experiment with an elliptical inclusion (009SC, see supplementary Figs. 8 + 9) was performed at 1.53 GPa confining pressure. Additionally, the piston-in experiments of Johannes and Puhan[29], the calibration used by Hacker and Kirby[10] and the compilation of Redfern et al.[30] is shown for comparison. We use the IC phase-transition calibration as a reference for the present work.

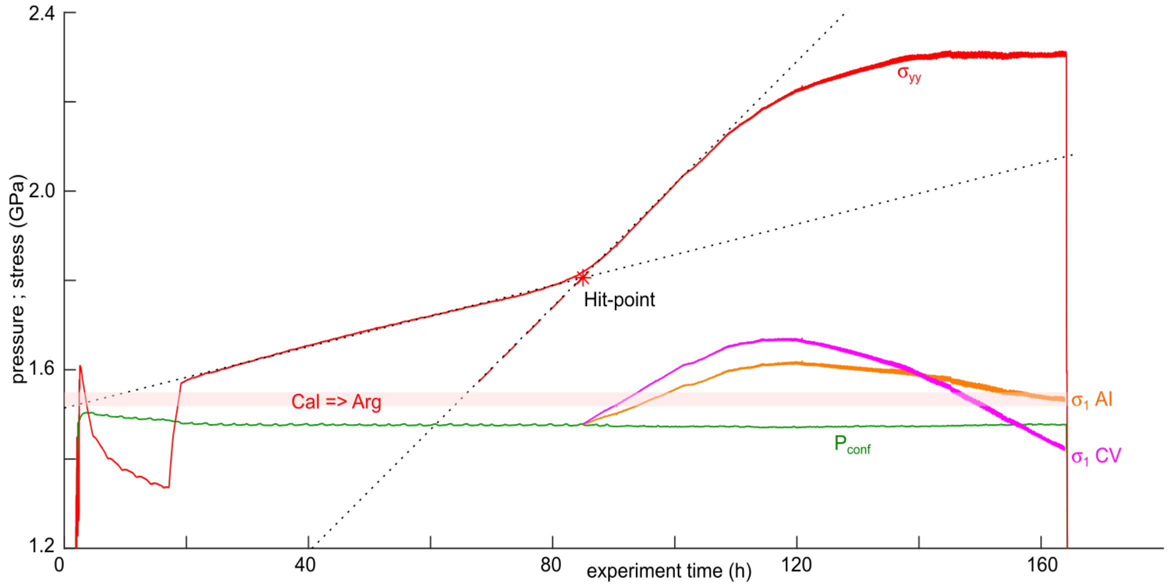

Fig. S2. Experimental stress-time curve for the experiment 010SC. Raw data for the experimental stress over time (red: deformation piston stress, σ_{yy} and green: pressure of the confining medium, P_{conf}) and two corrected σ_{yy}-curves. The correction applied for the magenta line (σ_1CV) assumes a constant volume cylinder that is progressively shortened (this correction is typically applied for coaxial Griggs apparatus experiments). Due to the volume conservation, the area increase is proportional to the shortening ($A_n = (A_0 \cdot l_0)/l_n$; where A is sample area and l is sample length). Up to a strain of 15–20% (accumulated at 120–135 h experiment time) this assumption holds but at later stages, the outer portion of the deforming material is mainly subject to hoop stress and does not support axial load as our model shows and was observed in various experiments[31]. We also performed an additional correction for our data by assuming an asymptotically increasing sample area (orange line, σ_1AI) after ref. [31] and references within. The faster increase of the area in the beginning (correction leads to lower σ_1) accounts for the barrelling effect. After a strain of ca. 15% (experiment time ca.120 h), the effective area is only marginally increasing as the added material is not supporting the axial load – thus σ_1 does not strongly decrease further.

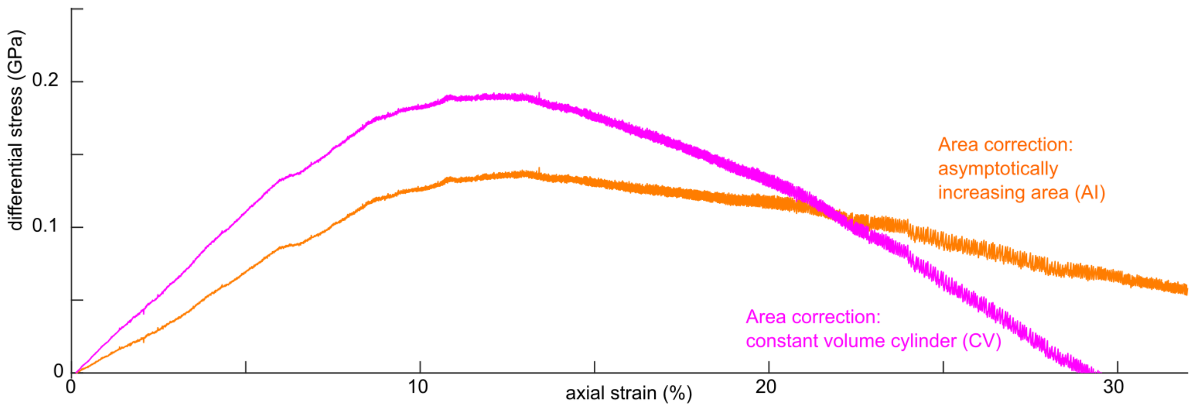

Fig. S3. Stress-strain curve derived from experiment 010SC. Depending on the applied correction, a peak differential stress of 0.14–0.18 GPa is reached. The strong decay of the differential stress for the classically used constant volume cylinder assumption after 15% axial strain is too high – this correction approach is shown for comparison to published data. As outlined in Fig. S1, assuming an asymptotically increasing sample area yields more realistic results, while underestimating peak differential stress in comparison to usual data treatment. Results achieved with both corrections up to a strain of 25% are in agreement with published rheology data for calcite[19,32].

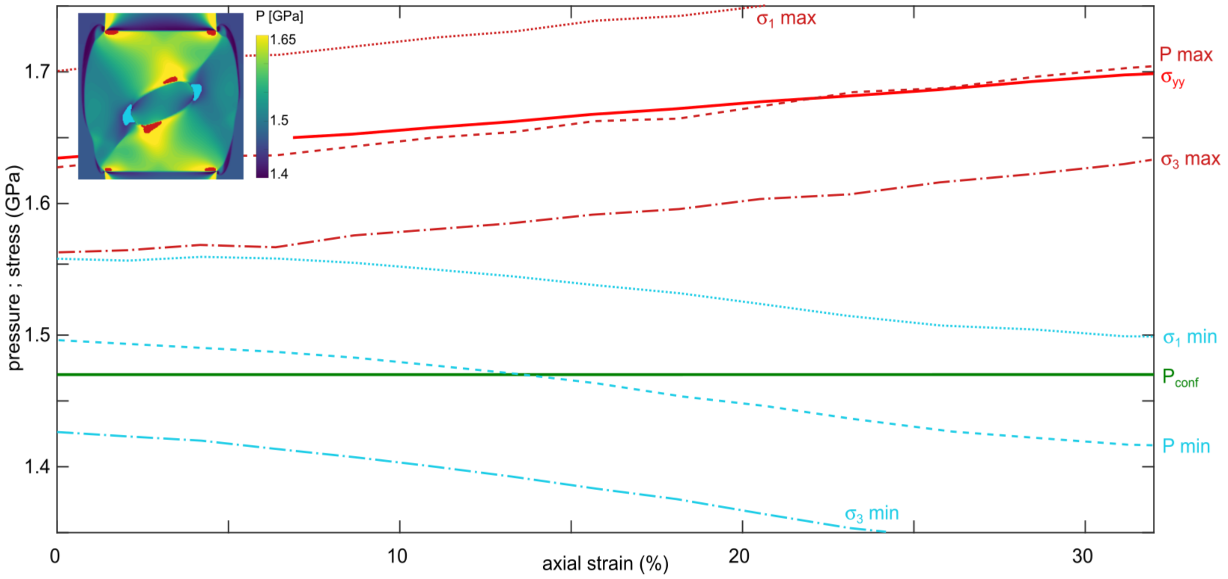

Fig. S4. Modelled stress-strain evolution. Stress-strain plot of modelled bulk principal stresses (solid lines, red and green), equivalent to the experimental curves (Fig. S1). The location where bulk stress values were sampled is shown in Figure 2 of the main text. In the inset, red and blue highlight the 1% fraction of pixels of all calcite material that experience maximum and minimum pressure values respectively. The thin lines in the plot represent the average of the principal stresses and pressure of the corresponding model points (pixels) for each time step.

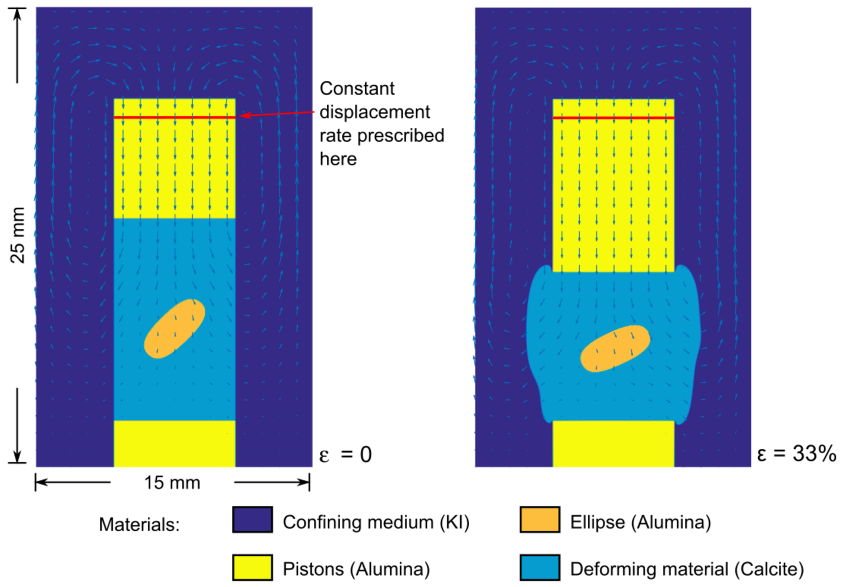

Fig. S5. Model materials and evolution. Material distribution in the model at the first and last time-step (axial strain, $\varepsilon = 33\%$).

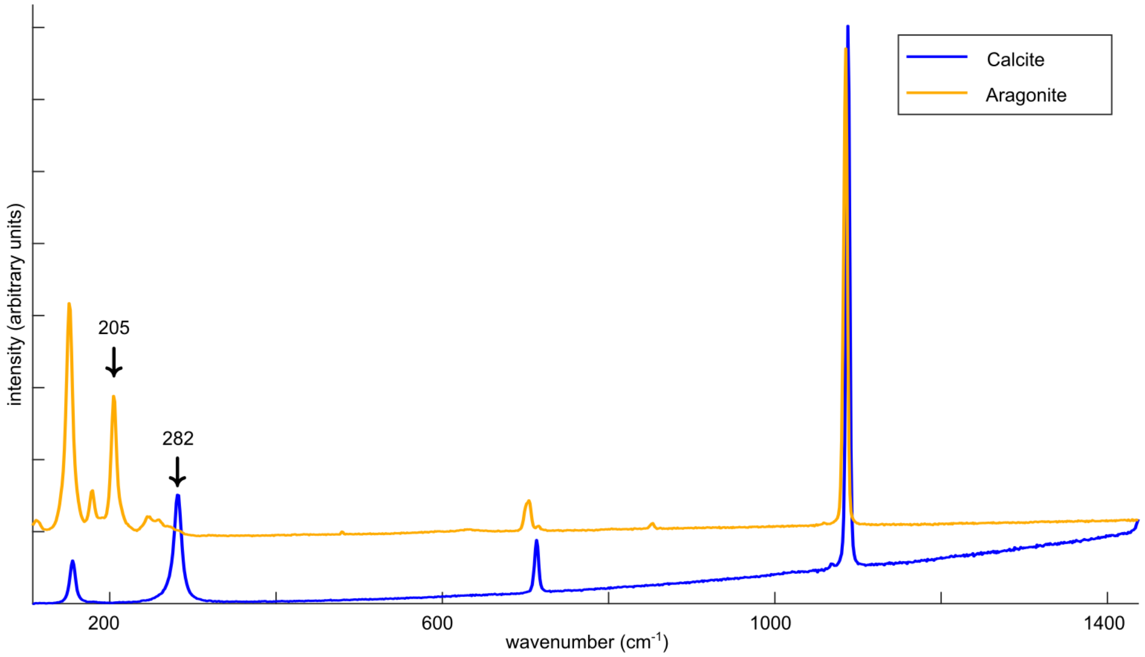

Fig. S6. Raman spectra of calcite and aragonite. Raman spectra of the analysed minerals, with indication of the peaks used to discriminate the material in the mapping.

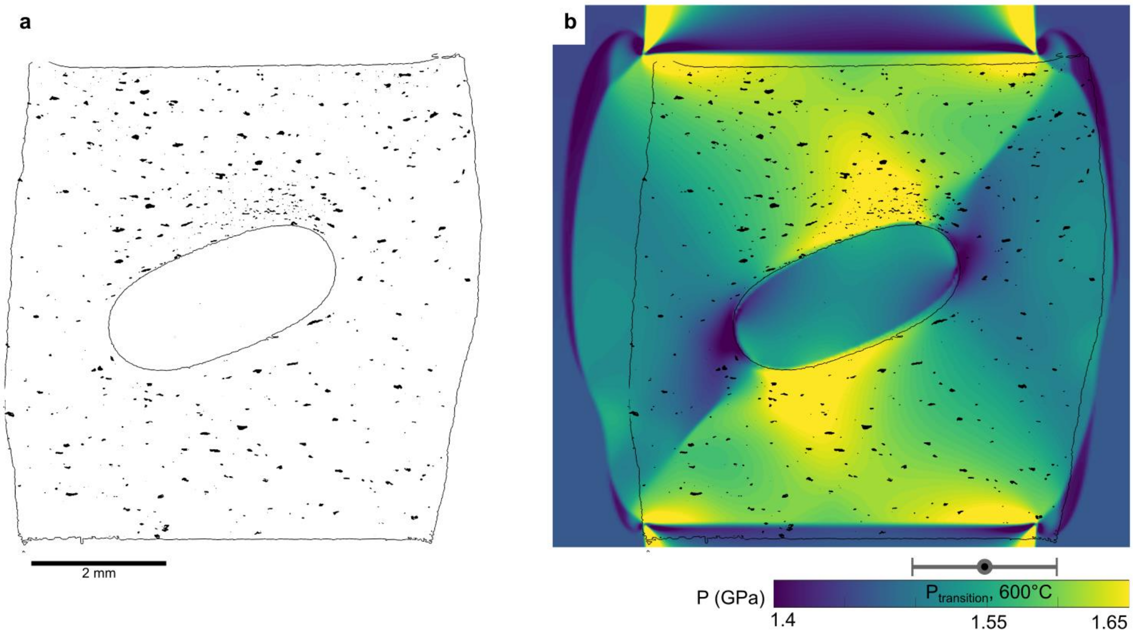

Fig. S7. Distribution of aragonite grains with size > 0.1 mm. a, An image derived from raw spectral data. Grains were selected by signal intensity of the aragonite 205 cm^{-1} peak. Afterwards the "erode" image cleanup procedure[33] was performed to remove small grains. **b,** Overlay of the image analysis result in a, on the numerical model presented in the main article. The phase transition pressure and experimental error is indicated in the legend.

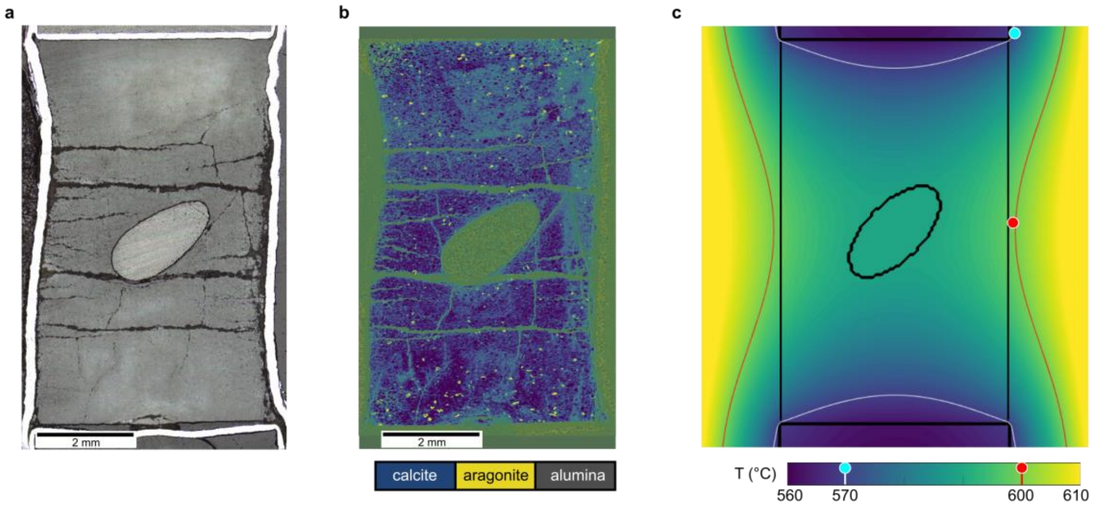

Fig. S8. Undeformed sample 009SC, hot-pressed at 1.53 GPa. a, Reflected light photomicrograph of the recovered sample 009SC. The horizontal cracks formed during unloading. **b,** Raman spectroscopy map of sample 009SC. The starting material, calcite, is shown in blue. The presence of aragonite is indicated by the yellow colour. An increased amount of aragonite is found close to the pistons, partially more towards the horizontal centre. **c,** Steady state temperature distribution model (2D) for this sample. The red dot represents the thermocouple positions at which the nominal experiment temperature is measured. The blue dot shows the position where the second thermocouple is placed in experiments where thermal gradients are determined. See Methods and the Supplementary Note for details.

Supplementary Note

Comparison of both experiments to the thermal model

In order to constrain the factors that could be responsible for the formation of aragonite, we also considered the effects of the heterogeneous temperature distribution. To do so, we calculated the temperature distribution within the sample (supplementary Fig. S8, c). Lower temperature and/or higher pressure favour the formation of aragonite. In the undeformed sample 009SC most of the aragonite is forming preferentially close to the pistons (upper part – corner regions). These variations can be explained either by lower temperatures or higher pressures in these regions of the sample. To investigate the effect of temperature gradients on the aragonite formation in all our experiments we calculated the 2D steady-state temperature distribution as it is shown in supplementary Fig. S8c. This model shows that the coldest region of the sample is next to the pistons, and more pronounced in the central part of the sample/piston interface. At the confining pressure of 1.53GPa (exp. 009SC) the phase transition is overstepped in the colder regions (<590°C) and therefore the temperature distribution could have been responsible the formation of aragonite close to the pistons in 009SC and partially also in 010SC. However, this effect cannot explain the aragonite formation adjacent to the ellipse in the deformation experiment 010SC. Furthermore, the amount of aragonite next to the pistons is higher towards the outer rims (warmer regions) in 010SC. These observations lead us to conclude that, the aragonite distribution of the deformed sample 010SC is in agreement with the pressure distribution (Fig. 3), whereas the aragonite distribution in the undeformed sample 090SC is controlled by the temperature field (without deformation no pressure variations are expected). Although temperature effects may influence the phase distribution in such experiments (e.g. 009SC), these are strongly overprinted by mechanical effects under deformation (see sample 010SC).

Calculation of the thermal model (supplementary Fig. S8, c)

To calculate the steady-state temperature distribution (supplementary Fig. S8, c), the temperature was set at the boundaries of the model (Dirichlet boundary conditions). Boundary conditions were chosen so that the temperature at the thermocouple position (red dot) is 600°C and a temperature gradient of 30°C is developed along the sample (up to blue dot). The temperature gradient is described in the Methods section.

The model has a resolution of 200x300 grid points and solves the steady-state temperature (T) distribution in a medium at rest for materials with different conductivity (k): div(k grad T) = 0[34]. The thermal conductivity for the materials in our experiment at 600°C is 12.6 W/(m K) for Al_2O_3[35], for calcite and the confining medium salt (KI) values were extrapolated and gave 2 W/(m K)[35,36] and 1.5 W/(m K)[35,37,38,39] respectively. Due to the uncertainty of extrapolation we tested different values (up to factor 2 difference) and the final result was qualitatively the same (i.e. the colder regions of the sample were located at the horizontal centre of the piston-sample interface).

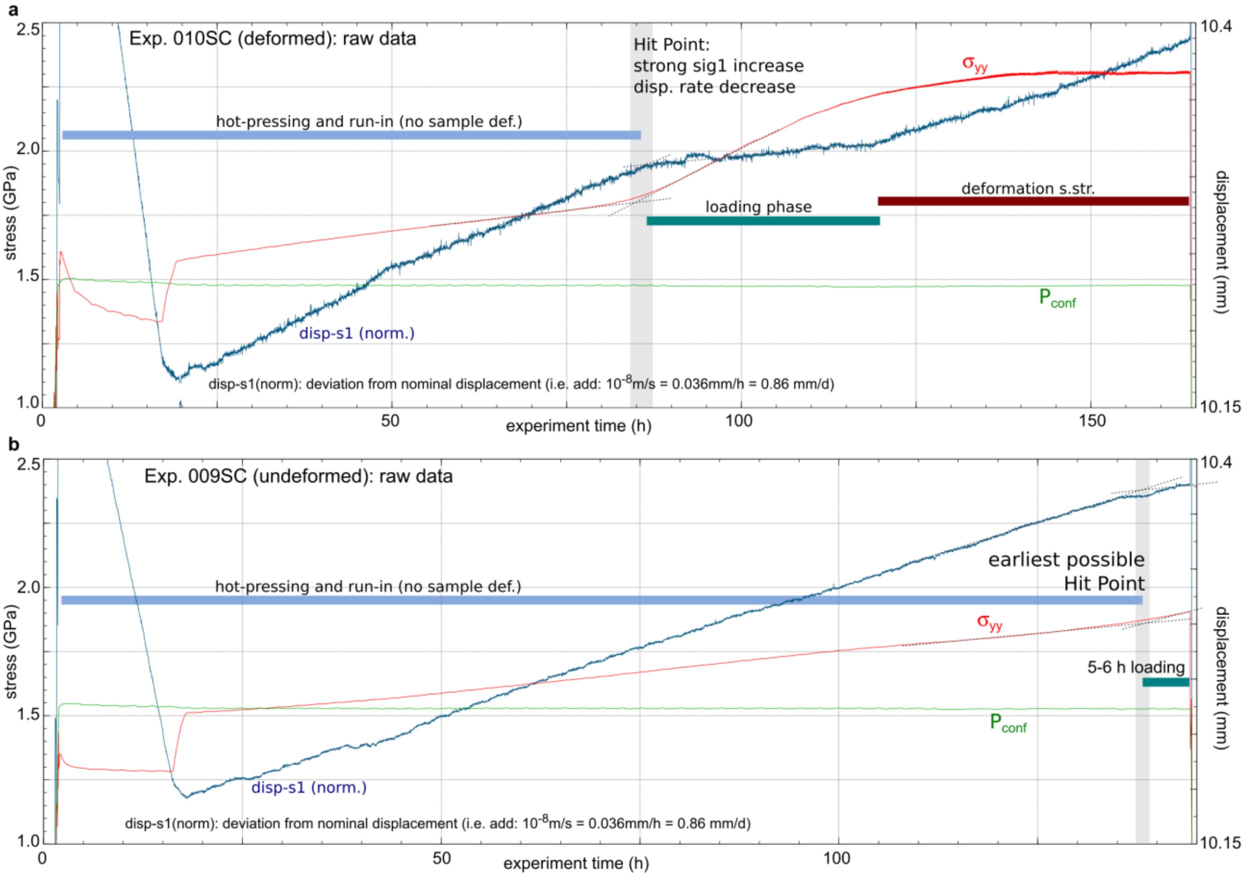

Fig. S9. Unprocessed stress and deformation data of experiments 010SC and 009SC. a) Unprocessed mechanical data for the deformation experiment 010SC with labels indicating different phases of the experiment. The plotted piston advance (disp-s1 norm) represents the deviation from the nominal velocity and shows changes in slope when different phases of the experiment are reached. The increase of σ_{yy} up to the hit-point is attributed to friction and not considered, as shown in supplementary Fig. S1. **b,** The dataset of 009SC shows, that the earliest possible hit-point occurred ca. 5-6 hours before quenching. During the period just after the hit-point (loading phase), deformation is distributed between the lead top-cap and the sample in varying amounts[12]. The maximal possible shortening was 0.2mm and the increase of σ_{yy} was 25 MPa. In experiment 010SC the loading phase prior to deformation lasted 35 hours (at equal deformation speed). 009SC, which experienced only 5-6 h loading and no visible deformation, is thus regarded to be an undeformed experiment. 009SC was subject to 135 hours of hot-pressing at a confining pressure of 1.53 GPa which is 0.05 GPa higher than 010SC.

Supplementary material references:

29. Johannes W., Puhan D., The calcite-aragonite transition, reinvestigated. *Contrib. to Mineral. Petrol.* **31,** 28–38 (1971).

30. Redfern S. A. T. , Salje E., Navrotsky A., High-temperature enthalpy at the orientational order-disorder transition in calcite: implications for the calcite/aragonite phase equilibrium. *Contrib. to Mineral. Petrol.* **101**, 479–484 (1989).

31. Omar T., Sadrekarimi A., Effects of multiple corrections on triaxial compression testing of sands. *J. Geoengin.* **9**, 75–83 (2014).

32 Renner J, Evans B, Siddiqi G., Dislocation creep of calcite. J*ournal of Geophysical Research: Solid Earth* **107.B12**, ECV-6 (2002)

33. Schindelin, J., *et al.*, "Fiji: an open-source platform for biological-image analysis", *Nature methods* **9(7)**, 676-682 (2012)

34. Landau, L. D. & Lifshits, E. M. *Fluid mechanics*. 198 (Pergamon Press, 1987).

35. Matweb, *Matweb Material Property Data,* http://www.matweb.com

36. Szelagowski, H., Arvanitidis, I. & Seetharaman, S. Effective thermal conductivity of porous strontium oxide and strontium carbonate samples. J. Appl. Phys. **85**, 193–198 (1999).

37. DDBST Gmbh, *Dortmund Data Bank,* http://www.ddbst.com/en/EED/PCP/HCP_C4911.php

38. Slack, G. A. & Ross, R. G. Thermal conductivity under pressure and through phase transitions in solid alkali halides: II. Theory. *J. Phys. C Solid State Phys.* **18**, 3957–3980 (1985).

39. McCarthy, K. A. & Ballard, S. S. Thermal Conductivity of Eight Halide Crystals in the Temperature Range 220°K to 390°K. J. *Appl. Phys*. **31**, 1410–1412 (1960).
